# Proteomic Biomarkers of Intrahepatic Cholestasis of Pregnancy

**DOI:** 10.1007/s43032-023-01437-z

**Published:** 2024-01-04

**Authors:** Weijian Zeng, Yanyan Hou, Wei Gu, Zheng Chen

**Affiliations:** 1 The International Peace Maternity and Child Health Hospital, School of Medicine, Shanghai Jiao Tong University, Shanghai, 200030 China; 2 Shanghai Key Laboratory of Embryo Original Diseases, The International Peace Maternity and Child Health Hospital, Shanghai, 200030 China; 3 Shanghai Municipal Key Clinical Specialty, The International Peace Maternity and Child Health Hospital, Shanghai, 200030 China

**Keywords:** Intrahepatic cholestasis of pregnancy, Proteomics, Ingenuity pathway analysis, PPARα, APOA2

## Abstract

Intrahepatic cholestasis of pregnancy (ICP) is a pregnancy-specific liver disease, which can lead to adverse fetal outcomes, including preterm labor and intrauterine death. The pathogenesis of ICP is still unclear. We hypothesized that pathological index leads to abnormal placenta changes in ICP. Investigation of these differences in protein expression in parallel profiling is essential to understand the comprehensive pathophysiological mechanism underlying ICP. The present study screened differentially expressed proteins (DEPs) as novel diagnostic markers for ICP. Proteomic profiles of placental tissues from 32 ICP patients and 24 healthy volunteers (controls) were analyzed. Our founding was valid by following western blotting and immunohistochemistry staining, respectively. The association of the key protein expression with clinicopathological features of ICP was further analyzed. A total of 178 DEPs were identified between the ICP and control groups. Functional enrichment analysis showed these proteins were significantly enriched in the PPAR singling pathway by KEGG and PPARα/RXRα activation by IPA. Apolipoprotein A2 (APOA2) was the only upregulated protein, which uniquely identified in ICP groups and related to both pathways. Validation of western blotting and immunohistochemical staining analysis showed significantly higher APOA2 expression in the ICP group than in the control group. Furthermore, the expression of APOA2 is associated with clinicopathological features in ICP groups. Receiver operating characteristic (ROC) curve analyses showed that the AUC of APOA2 was 0.8984 (95% confidence interval (CI): 0.772–1.000). This study has identified up-regulated APOA2 associated with PPAR singling pathway and PPARα/RXRα activation in ICP. Thus, APOA2 may be involved in ICP pathogenesis, serving as a novel biomarker for its diagnosis.

**Supplementary Information:**

The online version contains supplementary material available at 10.1007/s43032-023-01437-z.

## Introduction

Intrahepatic cholestasis of pregnancy (ICP) is one of the most common liver disorders during pregnancy, with a reported incidence rate ranging from 0.2 to 2% [1]. ICP typically occurs in the middle or late stages and rarely at the early stages of pregnancy [2]. ICP represents a serious threat to pregnant women and newborns and is associated with adverse pregnancy outcomes such as fetal distress, premature birth, and intrauterine death [3]. Ursodeoxycholic acid is the sole first-line treatment for ICP, via reducing bile acid levels and relieving pruritus symptoms [4]. However, several studies showed that Ursodeoxycholic acid treatment failed to produce a beneficial effect on bile acid levels or pruritus scores in ICP patients and did not alleviate adverse perinatal outcomes [5–7]. Therefore, there is a demand for additional therapeutic options in the management of ICP. Although there have been many advances in treatment strategies for ICP in recent years, the rate of adverse pregnancy outcomes remains high. The reason is that the pathophysiology involved in ICP progression remains largely unknown.

Protein expression patterns are indicators of developmental, physiological, or pathological states. Proteomics defined as the large-scale study of proteins is an important research area in the post-genomic era, which has a vital role in drug development as target molecules [8]. However, only a few studies have focused on the application of proteomics in the study of ICP.

In the present study, we applied proteomics to investigate on different protein expression profiles between ICP and normal pregnancy. These studies could provide useful and important information on the pathogenesis of ICP and identify new candidate therapeutic targets.

## Materials and Methods

### Study Design and Sample Collection

**Fig. 1:**
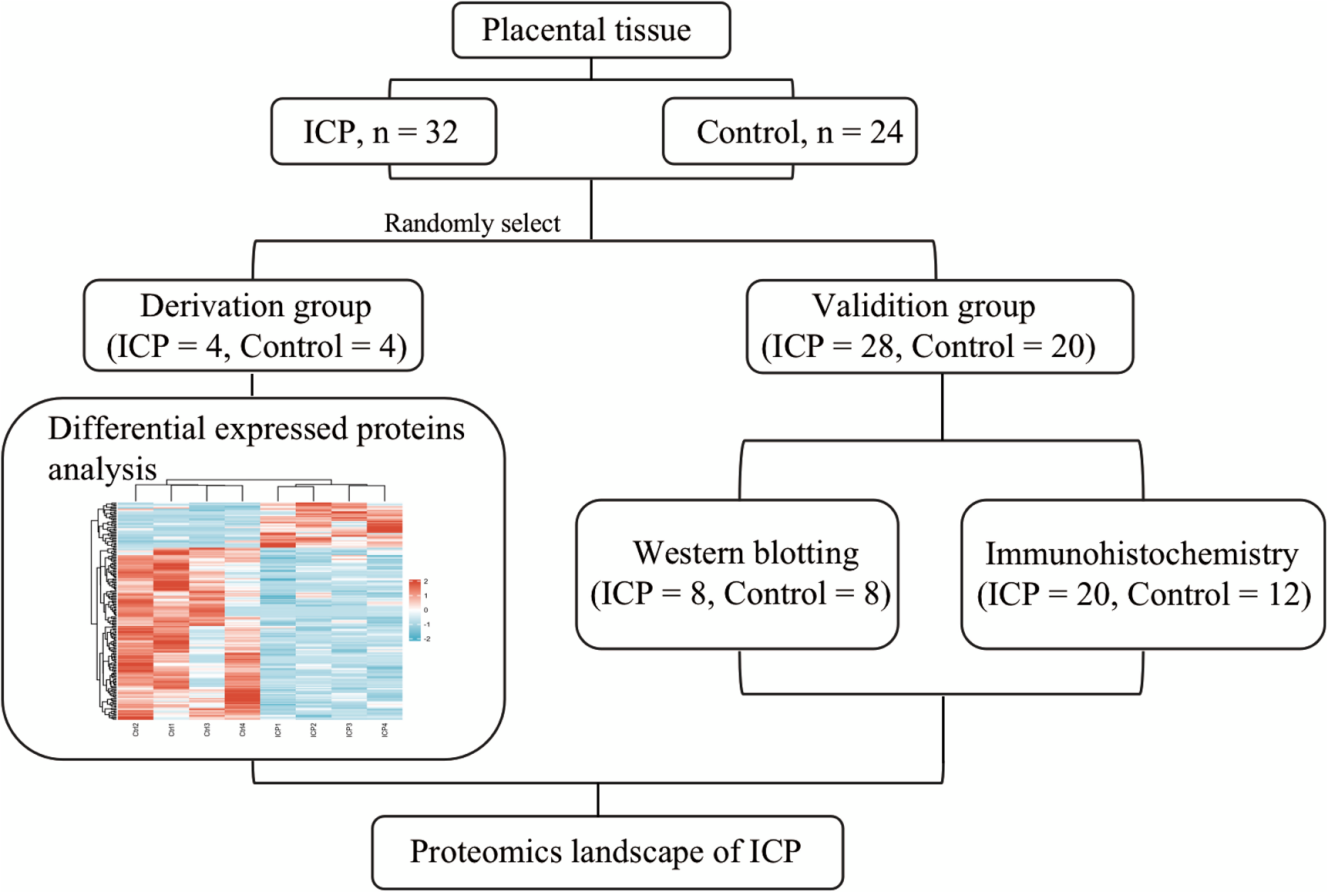
Overview of the study design and the workflow

We enrolled **32** pregnant women with ICP and **24** healthy controls who underwent cesarean section at the International Peace Maternity and Child Health Hospital (IPMCH) from March 2020 to September 2020. The inclusion criteria for patients with ICP were as follows: (1) Chinese nationality; (2) singleton pregnancy; and (3) diagnosis of ICP, presenting with pruritus and elevated liver transaminase and serum total bile acid levels. Patients with gestational diabetes mellitus, kidney disease, chronic hypertension, pregnancy with malignancy, or other diseases that cause liver dysfunction were excluded. The patients and healthy control subjects were randomly allocated into the derivation and validation groups (Fig. 1). All the collected placental tissues were stored at −80°C for further analysis. Four placental tissue samples from each group were randomly collected for proteome analysis. The rest placental tissues were selected for validation of our found with western blotting and immunohistochemistry, accordingly.

### Protein Extraction and Digestion

Eight samples (control groups, *n* = 4; ICP groups, *n* = 4) were randomly selected for protein profiling. Frozen samples were ground to a powder and homogenized in an extraction buffer containing 4% SDS, 0.1% PMSF, and 1× cocktail. After ultrasonication, the crude sample extracts were incubated at 95°C and centrifuged at 14,000 ×*g* for 30 min at 15°C. The supernatant was collected, and the Pierce BCA protein estimation assay kit (Pierce, Rockford, IL, USA) was used for quantification. The protein samples were precipitated by adding chilled acetone and incubated overnight. Subsequently, the samples were centrifuged at 15,000 ×*g* for 30 min at 4°C, and the supernatant was collected. Next, the samples were washed with 1 mL of cold acetone, vortexed two times, and resuspended in 50 μL of 8M urea. The supernatant was diluted with 50 mM Tris–HCl and digested at 37°C overnight after adding 40 mL of trypsin buffer. Subsequently, each filtration unit was centrifuged at 14,000 ×*g* for 30 min at 20°C, and digestion was terminated with 0.1% trifluoroacetic acid. Finally, after desalting with a C18 Stage Tip, the eluate was freeze-dried.

### Liquid Chromatography-Tandem Mass Spectrometry (LC-MS/MS)

The samples were analyzed on the Orbitrap Fusion Lumos (Thermo Fischer Scientific, San Jose, CA) mass spectrometer equipped with an LC column. Briefly, reverse-phase separation was performed with a gradient buffer from 5 to 90% buffer B (0.1% acetic acid) for 90 min at the flow rate of 600 nL·min^-1^. MS scans were captured in the peptide mode at the resolution of 120,000 and a scan range of 300–1400 *m/z*.

### Protein Quantification and Identification

MS raw files were processed by MaxQuant (version 1.5.3.20, Jürgen Cox, Max Planck Institute of Biochemistry, Martinsried, Germany), searching against the Uniport database of human protein sequences (12-2020,133,549 entries, downloaded from: http://www.uniprot.org) and the decoy database. Search parameters were set as follows: monoisotopic mass, peptide mass tolerance at ± 20 ppm and fragment mass tolerance at 0.1 Da, trypsin as the enzyme and allowing up to two missed cleavages. Variable modifications were defined as Oxidation(M) and Acetyl (Protein N-term), carbamidomethylation on cysteine were specified as fixed modifications. Protein identification was supported by at least one unique peptide identification. False discovery rate (FDR) of both proteins and peptides identification as set to be less than 0.01. Other parameters were set as default. Protein intensity values were used in the next quantification study. The missing values of certain proteins were replaced by 20% of the minimum positive values. Then, the intensity of each protein was normalized on the total intensity sum (normalized intensity values).

### Bioinformatics Analysis

Venn diagrams were generated with the R package VennDiagram (version 1.7.3) and *ggplot2* (version 2.3.6). DEPs between the ICP and control groups were screened using fold-change (FC) ≥ 2 and *P* value < 0.05 as the thresholds. Heatmaps and volcano plots of the DEPs were visualized with ComplexHeatmap (version 1.10.2) and *ggplot2* (version 3.3.3), respectively. Principal component analysis (PCA) of the DEPs was conducted with the R package (version 4.2.1) and *ggplot2* (version 2.3.6). Functional Annotation Tool DAVID Bioinformatics Resources 6.8 (https://david.ncifcrf.gov/) was used to elucidate the potential functions of the DEPs, upregulated proteins and downregulated proteins according to the biological process, cellular component, and molecular function categories of Gene Ontology (GO) annotations and Kyoto Encyclopedia of Genes and Genomes (KEGG) pathway, respectively. In addition, ingenuity pathway analysis (IPA, version 01-13, QIAGEN Redwood City, www.Ingenuity.com/) was applied to explore functional pathways and protein networks of DEPs. Right-tailed Fisher’s exact test was used to calculate a *P* value to determine the significance of each canonical pathway, and *P* values < 0.05 were considered statistically significant. Disease and functional protein networks analysis with DEPs were presented, along with a *z*-score. A *z*-score ≥ 2 or ≤− 2 was considered significant activation or significant inhibition, respectively.

### Western Blotting

Sixteen placental tissues (control groups, *n* = 8; ICP groups, *n* = 8) were lysed with RIPA lysis buffer (ASPEN) in the presence of protease inhibitor, and then, the quantification of the isolated protein was quantified and evaluated utilizing using the BCA Protein Assay Kit (Pierce, Rockford, IL, USA). The proteins were separated using sodium dodecyl sulfate-polyacrylamide gel electrophoresis and transferred to polyvinylidene difluoride membranes. Non-specific binding was blocked with 5% skimmed milk for 1 h at room temperature. Next, the membranes were incubated with anti-APOA2 (1:1000, Abcam, ab92478); anti-PPARα (1:1000, Abcam, ab227074) as the primary antibody at 4°C overnight, respectively, followed by a secondary antibody (1:5000, Affinity, Shanghai, China) at room temperature for 30 min. Beta-actin (1:2000, Abcam, ab8227) was used as an internal control. The antigens and antibodies were detected using high-signal ECL substrates, and the gray values of protein bands were analyzed and quantified using Image J.

### Immunohistochemistry Staining

Tissue immunohistochemistry (IHC) was performed with the rest placenta tissue enrolled (control group, *n* = 12, ICP group, *n* = 20). Paraffin-embedded placental tissue sections (thickness = 3 μm) were deparaffinized and rehydrated in aqueous ethanol solutions of different concentrations, followed by treatment with citric acid buffer to extract antigens. After washing with phosphate-buffered saline (PBS), endogenous peroxidase was blocked by incubating with 3% H_2_O_2_ for 10 min. PBS containing 10% goat serum was added to block non-specific binding. Next, the sections were incubated with anti-APOA2 (1:2000, Abcam, ab92478) overnight at 4°C, followed by a secondary antibody for 30 min at 20°C. The samples were incubated with an avidin-biotin complex reagent to detect the bound antibody, followed by diaminobenzidine solution for 2 h. The sections were then re-stained with hematoxylin. After rinsing and air-drying, the samples were sealed with neutral resin. Finally, the sections were examined under a fluorescence microscope and analyzed using Image-Pro Plus 6.0.

Two independent pathologists, who were blinded to the clinical and pathological data, evaluated the specimens. Sections were evaluated according to semi-quantitative immunoreactivity scores. We separately scored for the percentage of positive staining (0 = negative, 1 = 25%, 2 = 25–50%, 3 = 50–75%, and 4 = 75%) and the staining intensity (0 = none, 1 = weak, 2 = moderate, and 3 = strong). For each specimen, the summation of the two above gave the final score.

### Statistical Analysis

All data were analyzed using GraphPad 9.0 (San Diego, CA, USA). The data are presented as mean ± standard deviation (SD). Differences in the test between the two groups were compared using a *t*-test. Receiver operating characteristic (ROC) curve was constructed to assess the specificity and sensitivity of the IHC in ICP diagnosis by calculating the area under the curve (AUC). Statistical significance was set at *P* < 0.05.

## Results

### Patients and Clinical Characteristics

Clinical characteristics and perinatal outcomes of validation samples are summarized in Table 1. We found no significant difference between the two groups in terms of maternal age and the direct bilirubin (DBIL) level (both *P* >0.05). However, levels of total bile acids (TBA), total bilirubin (TBIL), alanine transaminase (ALT), aspartate transaminase (AST), and the maternal body mass index (BMI) were significantly higher in pregnant women with ICP than in healthy pregnant controls. While the maternal weight and the gestational age were significantly lower in pregnant women with ICP than in healthy pregnant controls.

**Table 1:** Clinical characteristics of all study participants

Parameter	Control (*n* = 24)	ICP (*n* = 32)	*P* value
Maternal age (years)	31.7 ± 5.0	31.5 ± 4.5	0.9160
Maternal BMI (kg·m^-2^)	22.2 ± 3.3	24.7 ± 3.8	0.0140
Gestational age (weeks)	38.5 ± 0.5	37.1 ± 1.1	0.0000
Maternal weight (g)	3303.5 ± 327.9	3120.6 ± 343.8	0.0496
TBA (μmol·L^-1^)	5.2 ± 2.3	31.8 ± 21.5	0.0000
TBIL (μmol·L^-1^)	8.4 ± 4.8	13.4 ± 11.3	0.0487
DBIL (μmol·L^-1^)	2.9 ± 1.1	5.4 ± 6.4	0.0651
ALT (U·L^-1^)	12.0 ± 4.7	47.9 ± 54.8	0.0002
AST (U·L^-1^)	15.3 ± 3.6	52.5 ± 71.8	0.0144

*BMI*, body mass index; *TBA*, total bile acids; *TBIL*, total bilirubin; *DBIL*, direct bilirubin; *ALT*, alanine transaminase; *AST*, aspartate transaminase

### Differentially Expressed Proteins Based on Proteomic Analysis

**Fig. 2:**
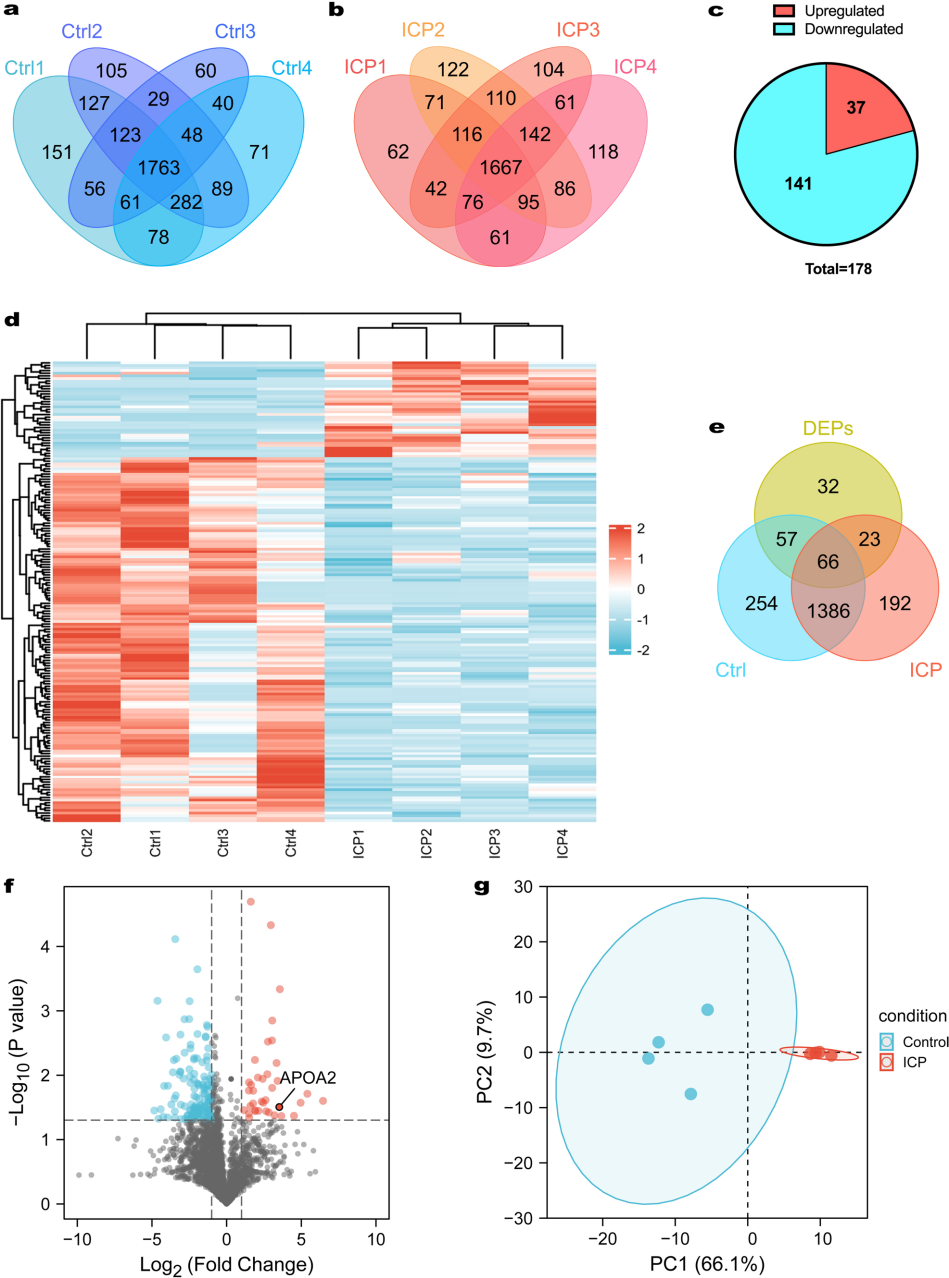
Identification of differentially expressed proteins (DEPs) between the intrahepatic cholestasis of pregnancy (ICP) and control groups. **a** Venn diagram for the distribution of identified proteins among the four samples in control groups. **b** Venn diagram for the distribution of identified proteins among the four samples in ICP groups. **c** The number of upregulated proteins (red) and downregulated proteins (blue) shown in a Pie chart. **d** Heatmap of DEPs between the control and ICP groups. Different colors represent the expression trends of DEPs between the two groups; upregulated and downregulated proteins are indicated in red and blue, respectively. **e** Venn diagrams showed the numbers of shared DEPs in control groups and ICP groups. **f** Volcano plot of all DEPs. Blue nodes represent 141 downregulated proteins and red nodes represent 37 upregulated proteins. **g** Principal component plot showing that the DEPs could clearly distinguish the ICP samples from controls.

To understand the difference in placental tissue at the proteomic level, we performed a proteomic analysis. 1763 proteins were all identified in the 4 individual samples in control groups (Fig. 2a). There were 1667 overlapping proteins among the 4 samples in ICP groups (Fig. 2b). Overall, a total of 3337 non-redundant proteins were identified in human placental tissues, and 3325 proteins with high confidence (FDR < 1%) were screened after normalization. Based on the standard of significance (FC ≥ 2 and *P* < 0.05), 178 DEPs were selected between the ICP and control groups, including 37 upregulated and 141 downregulated proteins (Fig. 2c), which were rendered as a visual heatmap (Fig. 2d). The top 20 DEPs and their gene names based on the highest fold-change expression are listed in Table 2. Fifty-seven and twenty-three DEPs were uniquely identified in control groups and ICP groups, respectively (Fig. 2e). In order to better demonstrate these differences between the two groups, a volcano plot **(**Fig. 2f**)** illustrating differential protein expression in placental tissue is shown. Meanwhile, PCA analysis indicated that the DEPs clearly distinguish the ICP samples from controls (Fig. 2g).

**Table 2:** Top 20 differentially expressed proteins (DEPs) between the intrahepatic cholestasis of pregnancy (ICP) and control groups

Accession number	Gene name	LogFC	*P* value	Regulated
Q9BSL1	UBAC1	6.456	0.0251	Up
Q9BXS6	NUSAP1	5.406	0.0194	Up
Q15848	ADIPOQ	4.955	0.0268	Up
P68402	PAFAH1B2	−4.844	0.0354	Down
Q13153	PAK1	−4.625	0.0007	Down
Q58EX2	SDK2	−4.565	0.0475	Down
O95674	CDS2	4.511	0.0427	Up
Q8IVN8	SBSPON	−4.413	0.0337	Down
Q8NHP6	MOSPD2	−4.242	0.0441	Down
P17152	TMEM11	−4.186	0.0147	Down
Q9Y5P6	GMPPB	−4.052	0.0026	Down
O14964	HGS	−3.944	0.0453	Down
O75427	LRCH4	−3.929	0.0146	Down
Q9Y2I8	WDR37	−3.700	0.0285	Down
P28347	TEAD1	3.667	0.0430	Up
Q9BPX5	ARPC5L	−3.596	0.0090	Down
Q14114	LRP8	3.565	0.0005	Up
Q8NBF2	NHLRC2	−3.534	0.0058	Down
P02652	APOA2	3.527	0.0312	Up
O43617	TRAPPC3	−3.488	0.0231	Down

### Functional Enrichment Analysis of DEPs

**Fig. 3:**
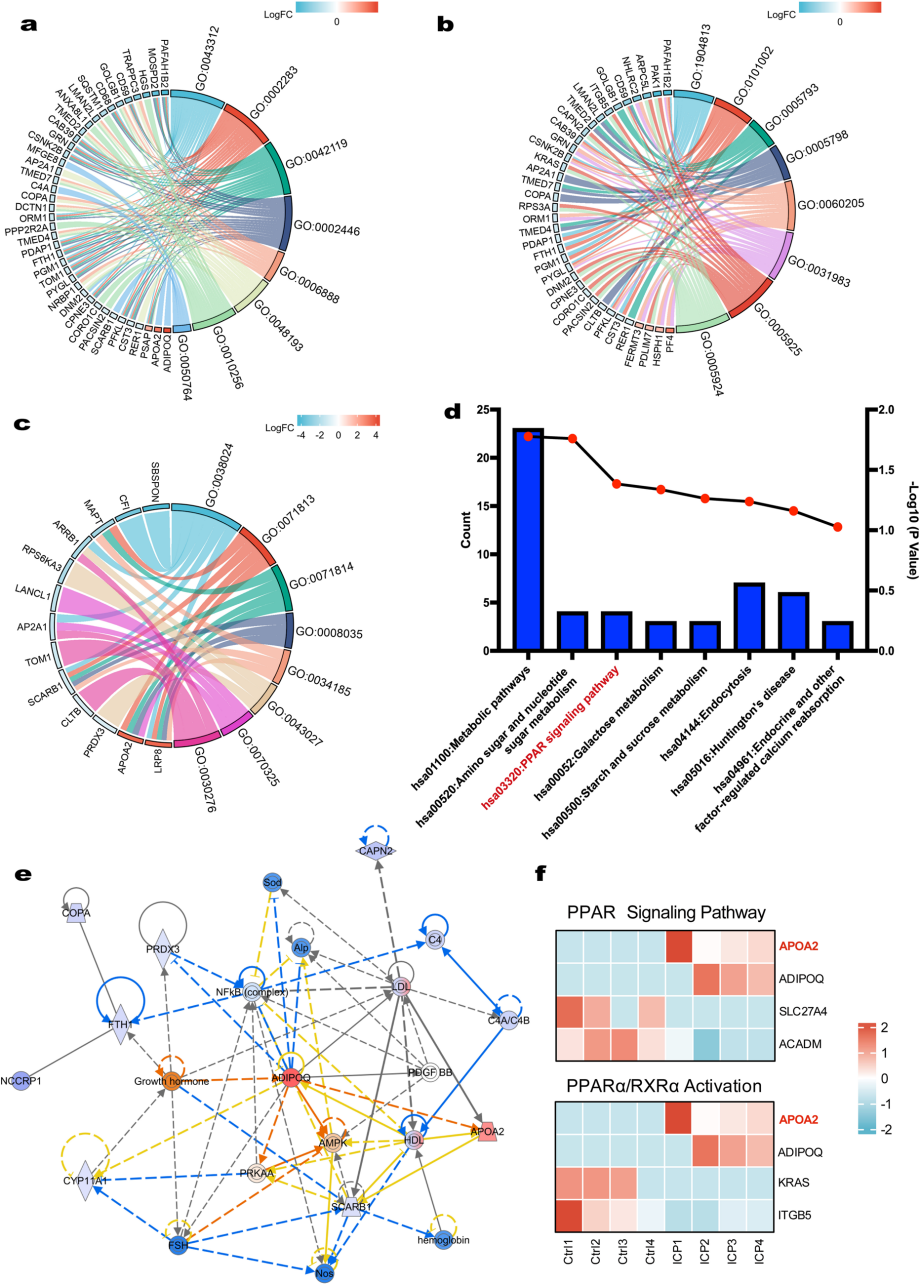
Functional enrichment analysis of differentially expressed proteins (DEPs). **a** The top eight significantly enriched Gene Ontology biological process (GO-BP). **b**–**c** Cellular component (CC), and molecular function (MF) terms. Upregulated and downregulated proteins are indicated in red and blue, respectively. **d** The top eight Kyoto Encyclopedia of Genes and Genomes (KEGG) pathways involve the identified DEPs. **e** Network of DEPs in placental tissue between the control group and ICP group associated with the “cellular compromise, inflammatory response, lipid metabolism” category by the IPA. Red labeling indicates the upregulated proteins and blue labeling indicates the downregulated proteins. Indirect relationships are represented by dotted lines and direct relationships by solid lines. **f** Expression levels of four DEPs in the PPAR signaling pathway and PPARα/RXRα activation measured by mass spectrometry, respectively

The potential biological functions of the DEPs were explored using GO and KEGG pathway analyses. GO enrichment analysis showed that the DEPs are involved in a wide range of biological processes, including neutrophil degranulation (GO:0043312), neutrophil activation involved in immune response (GO:0002283), and neutrophil activation (GO:0042119), neutrophil mediate immunity (GO:0002446), and regulation of phagocytosis (GO:0050764, Fig. 3a). The most abundant GO terms assigned in the cellular component category were ficolin-1-rich granule lumen (GO:1904813), ficolin-1-rich granule (GO:0101002), endoplasmic reticulum-Golgi intermediate compartment (GO:0005793), Golgi-associated vesicle (GO:0005798), and cytoplasmic vesicle lumen (GO:0060205, Fig. 3b). The proteins that participated in the molecular function were Cargo receptor activity (GO:0038024), lipoprotein particle binding (GO:0071813), protein-lipid complex binding (GO:0071814), high-density lipoprotein particle binding (GO:0008035), and lipoprotein particle receptor binding (GO:0070325, Fig. 3c). As shown in Fig. 3d, KEGG pathway analysis demonstrated that the DEPs were primarily involved in metabolism-related pathways, such as metabolic pathways (hsa01100), amino sugar and nucleotide sugar metabolism (hsa00520), the peroxisome proliferator-activated receptor (PPAR) signaling pathway (hsa03320), galactose metabolism (hsa00052), and starch and sucrose metabolism (hsa00500).

To better understand the DEPs relationships between the two groups, the data were further analyzed by the IPA Network Generation Algorithm and functional pathways. Ten networks were enriched and 6 of them with IPA score over 30 (shown in Additional file 1: Table S1). The most related lipid metabolism network emerged comprising 21 of those DEPs which is presented in Fig. 3e (IPA score 39). The network including nuclear factor kappa B (NF-κB) and apolipoprotein A2 (APOA2) was associated with cellular compromise, inflammatory response, and lipid metabolism. Top 8 canonical pathway analysis of DEPs by IPA is presented in Table 3. Integrin signaling, natural killer cell signaling, and NRF2-mediated oxidative stress response were enriched and indicated to be inhibited by IPA (*z*-score ≤ - 2). Otherwise, PPARα/RXRα activation was predicted to be activated with a *z*-score = 2. Collectively, based on our mass spectrometry data and bioinformatics analysis, the PPAR singling pathway by KEGG and PPARα/RXRα activation by IPA were enriched. Thus, the expression profiles of four DEPs involved with the PPAR singling pathway and PPARα/RXRα activation were presented, respectively (Fig. 3f). Interestingly, APOA2 was the only upregulated protein, which uniquely identified in ICP groups and could be found in the top 20 DEPs, that related to both pathways.

**Table 3:** Canonical pathway analysis of DEPs by IPA

Ingenuity canonical pathways	−log(*P* value)	Ratio	*z*-score	Molecules
Synaptogenesis signaling pathway	2.04E+00	2.24E-02	−1.89	AP2A1, ARPC5L, KRAS, LRP8, MAPT, PAK1, STXBP5
Integrin signaling	1.66E+00	2.35E-02	−2.236	ARPC5L, CAPN2, ITGB5, KRAS, PAK1
Actin cytoskeleton signaling	1.43E+00	2.04E-02	−1	ARPC5L, ITGB5, KRAS, PAK1, SSH3
PPARα/RXRα activation	1.25E+00	2.07E-02	2	ADIPOQ, APOA2, ITGB5, KRAS
Natural killer cell signaling	1.21E+00	2.01E-02	−2	COL1A1, HSPA4, KRAS, PAK1
NRF2-mediated oxidative stress response	9.91E-01	1.69E-02	−2	FTH1, KRAS, SCARB1, SQSTM1
Senescence pathway	7.40E-01	1.35E-02	−1	CAPN2, KRAS, PPP2R2A, SQSTM1
Breast Cancer Regulation by Stathmin1	3.44E-01	8.45E-03	−0.447	ADGRG1, KRAS, PAK1, PPP2R2A, RPS6KA3

Significance of the biological functional and disease terms were tested by the *P* value between DEPs and IPA knowledge base via right-tailed Fisher’s exact test. An activation *z*-score ≥ 2 indicates that the pathway is activated, while a *z*-score ≤ −2 indicates that the pathway is inhibited

**Fig. 4:**
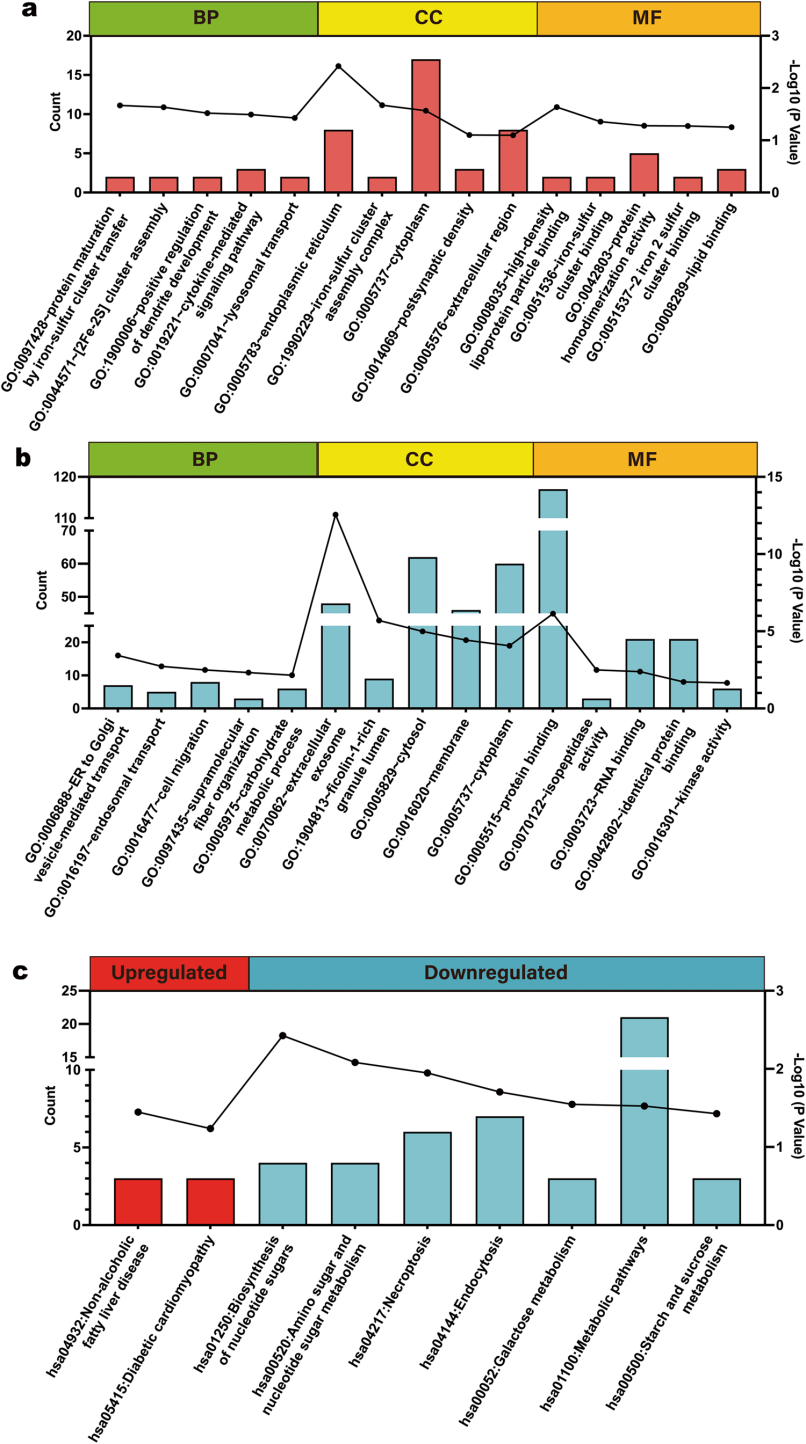
Functional enrichment analysis of upregulated proteins and downregulated proteins. **a** Top five significantly enriched Gene Ontology of biological process (BP), cellular component (CC), and molecular function (MF) terms involve upregulated proteins. **b** The top five significantly enriched Gene Ontology of BP, CC, and MF terms involve downregulated proteins. **c** The Kyoto Encyclopedia of Genes and Genomes (KEGG) pathways involve the of upregulated proteins and downregulated proteins

Furthermore, we performed GO enrichment analysis and KEGG pathway analysis on both upregulated proteins (*n* = 37) and downregulated proteins (*n* = 141), respectively. For upregulated proteins, the most abundant GO terms were protein maturation by iron-sulfur cluster transfer (GO:0097428), lysosomal transport (GO:0007041), endoplasmic reticulum (GO:0005783), iron-sulfur cluster assembly complex (GO:1990229), high-density lipoprotein particle binding (GO:0008035) and lipid binding (GO:0008289) were enriched with upregulated proteins according to (Fig. 4a**)**. For downregulated proteins, the most abundant GO terms were endosomal transport (GO:0016197), cell migration (GO:0016477), membrane (GO:0016020), extracellular exosome (GO:0070062), protein binding (GO:0005515), and isopeptidase activity (GO:0070122) (Fig. 4b). As shown in Fig. 4c, KEGG pathway analysis demonstrated that the upregulated proteins were primarily involved in non-alcoholic fatty liver disease (hsa04932) and diabetic cardiomyopathy (hsa05415), while the downregulated proteins were primarily involved in biosynthesis of nucleotide sugars (hsa01250), amino sugar and nucleotide sugar metabolism (hsa00520), and Necroptosis (hsa04217).

### Validation of Core Protein Expression by Western Blot and Immunohistochemistry (IHC) Analysis

**Fig. 5:**
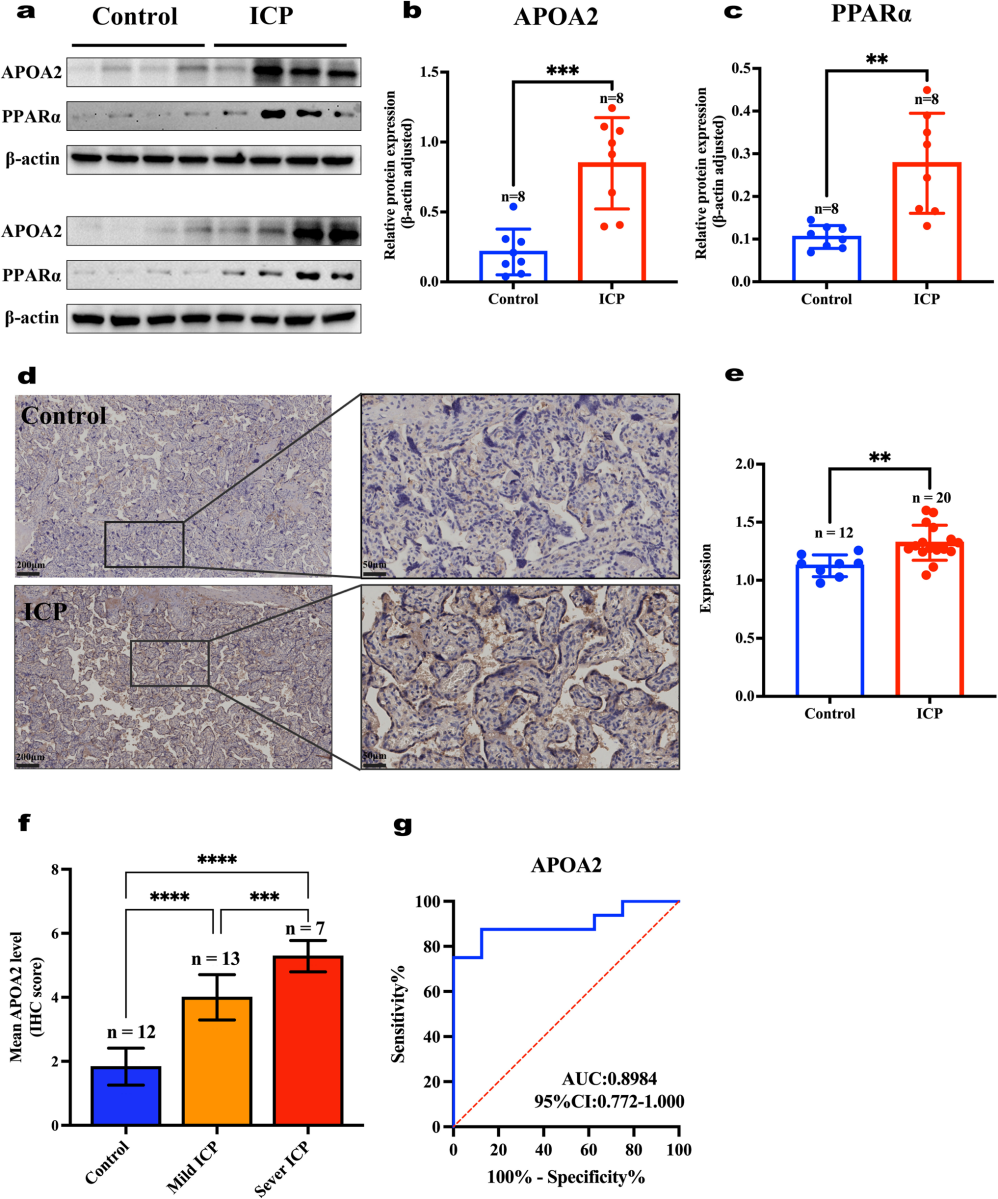
Validation of APOA2 protein expression. **a** Western blots. **b**–**c** Quantitative analysis showed that APOA2 and PPARα expression was higher in the ICP group (*n* = 8) than in the control group (*n* = 8), ***P < 0.001, **P < 0.01. **d** Representative image of APOA2 expression in placental tissues from the ICP and control groups detected using immunohistochemical (IHC) staining. **e** Quantitative analysis showed that APOA2 expression was higher in the ICP group (*n* = 20) than in the control group (*n* = 12). **P < 0.01. **f** Immunohistochemistry scores (IS) of APOA2 in control (TBA < 10, *n* = 12), mild ICP (TBA: 10 ~ 40, *n* = 13), and severe ICP placental tissues (TBA ≥ 40, *n* = 7), ****P< 0.0001, ***P < 0.001. **g** ROC curves of the APOA2 protein

Combined with bioinformatics analysis, the PPAR singling pathway and PPARα/RXRα activation that enriched pathways could be related to the mechanism of ICP. Given the pivotal role of PPARα in liver lipid homeostasis and the treatment of fenofibrate, severe as a PPARα agonist, against cholestasis-induced hepatic injury [6], the expression of APOA2 and PPARα was detected by western blotting to verify the relationship between ICP and control groups. As shown in Fig. 5a–b, APOA2 protein expression was markedly higher in the ICP group (*n* = 8) than that in the control group (*n* = 8, *P* < 0.001). Similarly, the expression of PPARα was also significantly higher in the ICP group (*n* = 8) than in control group (*n* = 8, *P* < 0.01, Fig. 5a and c). In addition, IHC analysis of fixed and paraffin-embedded placental tissue for validation of ICP proteomics data and western blotting results confirmed the presence of APOA2 in the ICP groups, while negative controls showed little signals (Fig. 5d). APOA2 immunostaining was absent or weak in normal placental tissues but moderate-to-strong in ICP groups. As shown in Fig. 5e, the expression of APOA2 in ICP groups (*n* = 20, *P* < 0.01) was significant higher. Consistent with our proteomics and western blotting analysis.

### Clinical Significance of APOA2

We further investigated the association between APOA2 expression and clinicopathological features in ICP groups. The results showed that increased APOA2 expression was significantly associated with maternal age (*P* = 0.0135) and the level of TBA (*P* = 0.0002), but not with patient BMI, TBIL, ALT, or AST level (Table 4). Also, the APOA2 expression has no effect on pruritus symptoms, mode of delivery and Apgar score. Furthermore, the IHC score significantly correlated with the expression of APOA2 and the degree of ICP (Fig. 5f). The IHC score was significantly higher with the increased APOA2 levels among the control group (*n* = 12), mild ICP group (*n* = 13) and severe ICP group (*n* = 7), accordingly. In addition, ROC curve analyses showed that the AUC of APOA2 was 0.8984 (95% confidence interval (CI): 0.772–1.000), indicating that APOA2 may serve as a potential biomarker for ICP (Fig. 5g).

**Table 4:** The correlation of APOA2 expression with clinicopathological features in ICP

	Cases (*n*)	APOA2 IS	*P* value
		Mean ± SD	
Age (years)			
<35	16	4.8 ± 0.9	0.0135
≥35	4	3.5 ± 0.6	
BMI (kg·m^-2^)			
<28	15	4.7 ± 1.0	0.1798
≥28	5	4.0 ± 0.7	
TBA(μmol·L^-1^)			
10~40	13	4.0 ± 0.7	0.0002
≥40	7	5.4 ± 0.5	
TBIL(μmol·L^-1^)			
5–21	17	4.4 ± 0.9	0.0987
>21	3	5.3 ± 1.2	
ALT(U·L^-1^)			
<35	12	4.3 ± 0.8	0.1526
≥35	8	4.9 ± 1.1	
AST(U·L^-1^)			
<35	12	4.4 ± 0.7	0.6422
≥35	8	4.6 ± 1.3	
Pruritus			
Yes	14	4.5 ± 0.9	0.9999
No	6	4.5 ± 1.2	
Normal delivery			
Yes	5	3.8 ± 0.8	0.0532
No	15	4.7 ± 0.9	
Apgar score			
<7	1	3.0 ± 0.0	0.1050
≥7	19	4.6 ± 0.9	

*BMI*, body mass index; *TBA*, total bile acids; *TBIL*, total bilirubin; *DBIL*, direct bilirubin; *ALT*, alanine transaminase; *AST*, aspartate transaminase. Significance of difference (*P* value) between categories was analyzed by unpaired *t* test. *P <* 0.05 for the significance of difference

## Discussion

ICP is reversible cholestasis typically onsetting during mid or late pregnancy, characterized by the symptoms of pruritus and elevated serum bile acid levels [9]. The etiology of ICP is multifactorial and may be related to elevated estrogen levels and altered hepatobiliary transporter protein expression during pregnancy. In the Jelski et al. study, the elevation of class I alcohol dehydrogenase isoenzyme (ADH I) released from damaged liver cells in ICP patients may increase the total alcohol dehydrogenase (ADH) level in serum which is positively correlated with ADH I [10]. Further investigation validated the predictive diagnosis value of both ADH and ADH I in serum means that ADH (especially ADH I) could serve as a potential diagnostic ICP marker [11]. Although different tentative studies about ICP have been carried out, the precise pathogenesis of ICP still remains unclear [12]. Placenta is a critical organ for maintaining the development and growth of the fetus in utero, which is the only medium for the exchange of nutrients and oxygen between mother and fetus during the entire pregnancy [13], but usually discarded after delivery. Evidently, the recent advances in proteomics technologies provide us with a global and unbiased scan of the proteome under investigation[8]. Thus, placental tissues were collected and tested for more possible DEPs by proteomics analysis. In our study, more proteins in the placental tissue were identified with higher resolution mass spectrometry, which is 3337, more DEPs including 37 upregulated and 141 downregulated proteins were found. Compared with He et al. [14] study, only 37 DEPs were identified in the placental tissue of ICP patients compared with normal controls by the method of two-dimensional electrophoresis and mass spectrometry. In Zhang et al. study [15], only 2120 proteins were identified in the placental tissue of ICP patients compared with normal controls by isobaric tags for relative and absolute quantification-based proteomics approach, including 29 upregulated proteins and 9 downregulated proteins. In Zou et al. study [16], the serum proteomes of pregnant women with ICP and healthy pregnant women were tested by data-independent acquisition proteomics technology. 615 proteins were identified and 526 proteins were quantitatively analyzed. Given that the incidence of ICP varies with ethnicity and geographic location [7], individual differences may also serve as possible explanations for the different proteins detected. Therefore, the more DEPs that we screened were conducted with the following bioinformatics analysis for further exploration.

ICP complications are associated with the immune system. The impairment of immunomodulatory networks may inhibit fetal and placental growth [17]. Zou et al. [16] found that hub proteins associated with ICP primarily affect neutrophil activation involved in the immune response. Previous studies found neutrophil count decreased in patients with ICP [18] and decreased neutrophil count may serve as a useful supplementary index in ICP diagnostic algorithms [19]. Our functional enrichment analysis revealed that the identified proteins were primarily enriched in neutrophil activation involved in immune response and neutrophil degranulation. Elevated bile acid levels damage hepatocytes which mediates the recruitment of immune cells, such as neutrophils to the liver [18]. Consistent with our study, we observed that the identified DEPs were also enriched in neutrophil-related functions. Furthermore, a previous study showed that ICP is associated with changes in metabolic syndrome, particularly impaired glucose tolerance and dyslipidemia [20]. In the present study, our analysis revealed that the detected DEPs are also involved in sugar metabolism-related pathways.

The bile acid receptors FXR and TGR5 are involved in glucose homeostasis. Decreased activity of these receptors and elevated levels of circulating bile acid may contribute to altered glucose metabolism in pregnant women with ICP [21]. Studies showed that bile acids recognized with G protein-coupled bile acid receptor 1 could induce NF-κB pathway activation, consequently upregulated inflammatory genes in trophoblasts, leading to inflammation and aberrant leukocyte infiltration in placenta [22]. Peroxiredoxin-3 (PRDX3) could protect trophoblast cells against cellular senescence and mitochondrial dysfunction induced by oxidative stress [23]; the downregulated PRXD3 found in our study could be the reflection of oxidative stress-induced inflammation. The NF-κB and PPAR-γ were upregulated in both placental tissues of patients with ICP and taurocholate acid induced HTR8/SVneo cells [24]. PPARα, PPARδ, and PPARγ are three transcription factors of the PPARs family, which are involved in glucose and lipid metabolism and have anti-inflammatory effects [25]. In our study, the IPA Network enriched that NF-κB complex was associated with inflammatory response and lipid metabolism. PPARα/RXRα activation was predicted to be activated by IPA. In addition, the expression of APOA2 and PPARα was both higher in ICP groups compared with control groups.

The human apolipoprotein gene family consists of 22 members; Apolipoprotein A1(APOA1) and APOA2 were two of them [26]. PPARα regulates the synthesis of APOA1 and APOA2 that participate in high-density lipoprotein (HDL) particle formation [27]. Study showed that expressing APOA2 and APOA1 are more vulnerable to suffering from atherosclerosis than those expressing APOA1 alone in mice [28]. APOA2 could affect HDL metabolism and stabilize HDL structure by its association with lipids. APOA2-containing HDL particles tend to be larger and possess less antioxidant activity [29]. Additionally, APOA2 could catalyze two-electron reductions of lipid hydroperoxides to their corresponding hydroxides, which could lead to membrane damage caused by lipid peroxides and promote lipid peroxidation pathway activity[30]. The formation of specifically oxidized forms of APOA2 and its oxidation of methionine residues was related to the reduction of redox-active phospholipid hydroperoxides [31, 32]. Thus, highly expressed APOA2 in the placenta could be a sign of PPAR singling pathway and PPARα/RXRα activation; then, the upregulated APOA2 cause severe oxidative damage and trigger a series of inflammatory responses consequently.

The study of APOA2 mainly focused on hepatocellular carcinoma, prostate cancer, gastric cancer, myeloma, and pancreatic cancer [26], however, the importance of APOA2 on obstetrics and gynecology was reported recently. In women with gestational diabetes, APOA2 is significantly correlated with gestational age and may serve as a potential biomarker for preterm birth [33]. Another study found that suboptimal maternal nutrition is associated with altered expression of cholesterol transport-related genes, such as *APOA2*, which may affect fetal development and pose a risk of disease later in life [34]. In our study, APOA2 was first reported that could be related to the mechanism of ICP. Additionally, the ROC of APOA2 suggested that APOA2 could effectively distinguish ICP samples from controls. Thus, combined with bioinformatics analysis and experimental verification, APOA2 could be a diagnosis index for ICP and a potential candidate to contribute to ICP pathogenesis.

Finally, the present study has several limitations. First, the sample size was small. In the future, we intend to prospectively recruit more patients with ICP to validate our findings. In addition, we only evaluated APOA2 levels in the present experiment but did not examine the way of highly expressed APOA2 affects the mechanism in ICP. We will continue to explore the relationship between APOA2 with ICP in vitro and in vivo in subsequent work.

## Conclusion

We investigated the differences in proteomic profiles between women with ICP and those with normal pregnancies and found APOA2 as a promising and potentially effective diagnostic biomarker for ICP. Collectively, our study may bring a new insight for upcoming new detection strategies of ICP thus helping the establishment of novel therapeutic targets for this disease.

### Supplementary Information

ESM 1

## Data Availability

The original contributions presented in the study are included in the article. Further inquiries can be directed to the corresponding author. The mass spectrometric proteomics data are deposited into the Proteome Xchange Consortium (http://proteomecentral.proteomexchange.org) via the iProX partner repository (dataset identifier: PXD033701).
